# Perceptions and Attitudes Towards Clinical Pharmacy Services and Their Impact on The Management of Cancer in Taif, Saudi Arabia

**DOI:** 10.31557/APJCP.2020.21.2.531

**Published:** 2020

**Authors:** Ahmed M Kabel, Morouj M Bakr, Abeer M Alshanbari, Shahad M Alwagdani, Hanan A Altalhi, Shayma H Alzaidi, Meaad H Altowairqi

**Affiliations:** 1 *Department of Clinical Pharmacy, *; 1 *1Pharm D, College of Pharmacy, Taif University, Taif, Saudi Arabia, *; 2 *Department of Pharmacology, Faculty of Medicine, Tanta University, Tanta, Egypt, *; 4 *PharmD candidate, Medical University of South Carolina, USA *

**Keywords:** Cancer, clinical pharmacy, students, healthcare providers, perception

## Abstract

**Aim:**

To assess the perception and attitude of HCPs and health-related science colleges’ students regarding the clinical pharmacists’ roles and responsibilities in providing better pharmaceutical care to patients in Taif, Saudi Arabia and to detect its impact on management of cancer.

**Methods:**

This study was conducted in four randomly selected hospitals in Taif and three health-related science colleges in Taif University. A questionnaire was distributed to HCPs and another questionnaire to students of health-related science colleges.

**Results:**

Three quarters of students perceived that the clinical pharmacist is an important part of the healthcare team. Two-thirds of HCPs expressed confidence in the ability of clinical pharmacists to minimize medication errors. Although two-thirds of HCPs reported that they did not have clinical pharmacists in their institutions, there was substantial willingness among HCPs to cooperate with the clinical pharmacists. Most HCPs expressed the view that the clinical pharmacist is an important integral part of the healthcare team and has a positive impact on cancer management.

**Conclusion:**

HCPs and students of health-related science colleges valued the role of clinical pharmacists in healthcare delivery and management of cancer. However, new developments in clinical pharmacy services in Taif hospitals are recommended to improve perception and attitudes towards the clinical pharmacy services. Also, well-organized programs should be conducted to students of health-related science colleges to improve their perceptions and attitudes towards the clinical pharmacy services which may have a positive impact on cancer management.

## Introduction

Cancer is a group of diseases characterized by unregulated cell growth and differentiation. Management of cancer necessitates effective cooperation between various healthcare services and healthcare providers including the physicians, nurses and the clinical pharmacists (Taplin et al., 2015). Clinical pharmacy is a health science whereby pharmacists provide patient care that optimizes medication therapy and promotes health, wellness and disease prevention (Francis et al., 2014; Rotta et al., 2015). This field of pharmacy practice focuses on patient-oriented rather than drug product-oriented service (Kelly et al., 2013). This science emerged as a result of dissatisfaction with old practice norms and the pressing need for a competent health professional with a comprehensive knowledge in the therapeutic use of drugs. Clinical pharmacists are a primary source of scientifically valid information and advice regarding the safe, appropriate, and cost–effective use of medications (Alhamoudi et al., 2018; Shim et al., 2018). Already, the level of interaction between the physicians and pharmacists in the developed world is high, resulting in safer, more effective and less costly drug therapy (Asiri, 2011; Saddique, 2011; Al-Qadheeb et al., 2012).

In 1959, the first college of pharmacy in Saudi Arabia was established and baccalaureate degrees in pharmacy were granted. In mid-1970s, clinical pharmacy practice was introduced in the country (Alhamoudi et al., 2018). After thirteen years, the first pharmacy department in King Khalid University Hospital (KKUH) implemented clinical pharmacy program outside the United States of America (USA) (Saddique, 2011). About 15 clinical pharmacists after the initial trial were employed, and then the service was expanded to cover all hospitals. In Saudi Arabia, clinical pharmacy is relatively well developed (Asiri, 2011). Whereas the number of academic centers are currently 28 academic centers that grant pharmacy degrees, some centers offer a Bachelor in Pharmacy (B-Pharm) and others offer the Master-level (MSc) or Doctor of Pharmacy (PharmD) degrees. Most universities are headings for clinical pharmacy, which hints will eventually be phased out from B-Pharm degree. In some universities, students start in the B-Pharm program. If they meet specific criteria during their studies, they will progress into the PharmD program (Al-Qadheeb et al., 2012; Bin Saleh et al., 2015). The aim of this study was to assess the perception and attitude of HCPs and health-related science colleges’ students regarding the clinical pharmacists’ roles and responsibilities in providing better pharmaceutical care to patients in Taif, Saudi Arabia and to detect its impact on the management of cancer. 

*Subjects and methods*

A cross-sectional study was conducted in four randomly selected hospitals (King Faisal Hospital, King Abdul-Aziz Hospital, Pediatrics Hospital and Prince Mansour Military Hospital) and three health-related science colleges in Taif University (College of medicine, college of applied health sciences (AHS) and college of nursing). This study was conducted according to the National Research Council Guidelines and was approved by the ethics committee of Taif University, Saudi Arabia (Code 40-35-0181). The participants were randomly selected from lists provided by their facility administrators. A written consent was obtained from the participants before being included in this study. 

*Inclusion criteria*

1. Male and female students of health-related science colleges in Taif University (College of medicine, college of AHS and college of nursing).

2. Male and female HCPs including physicians, nurses and technicians working in physiotherapy, laboratory and radiology departments in King Faisal Hospital, King Abdul-Aziz Hospital, Pediatrics Hospital and Prince Mansour Military Hospital in Taif, Kingdom of Saudi Arabia (KSA).

*Exclusion criteria*

1. Male and female students of other colleges in Taif University.

2. Male and female HCPs in departments other than the above-mentioned.

## Materials and Methods

*Methodology*

The questionnaires were distributed to HCPs and students of health-related science colleges. The participants were approached directly to arrange a 15 minutes interview with the researchers at a convenient time. The questionnaires were completed by the participants under the supervision of the researchers in order to improve clarity and limit response bias. 

The questionnaire consists of a series of questions prepared by the researchers with one version targeted at HCPs and the other at students. To ensure face validity, the questionnaire was sent to three academics and three physicians with a wide range of professional experience. Their views and comments were considered and then incorporated, where appropriate, into the final version of the questionnaire. To assess test-retest reliability, the questionnaire was administered on two occasions to 12 randomly selected HCPs. The second testing took place two weeks later. Test-retest reliability was calculated using Spearman’s correlation coefficient (r). The rho-value was 0.82, which implies acceptable test-retest reliability (Al-Qadheeb et al., 2012). 

 Respondents were asked to answer a question using the options “yes” or “no”, or to rate their response using the options “agree”, “neutral”, or “disagree”. The study was carried out over a period of three months (October to December 2018).

*Statistical analysis*

The statistical analysis of the results was carried out using Minitab 16 (Minitab Statistical Software, State College, PA, Research Resource Identifier: SCR_014483). Descriptive analysis was used to calculate the proportion of each group of respondents who agreed/disagreed with each statement in the questionnaire. Chi-square test was used to identify any significant difference among the participants’ responses regarding certain statements in the questionnaire. P-values less than 0.05 were considered statistically significant.

## Results

*Demographic data of the students*

Table 1 shows the demographic data of students who gave their opinions in response to the questions included in the questionnaire. The respondents were 79 (22.8 %) male and 267 (77.2 %) female. 83 (24%) of the respondents were less than 20 years old, 258 (74.6 %) were from 20 to 25 years old and 5 (1.4%) were older than 25 years old. Among them were 186 (53.8 %) from the college of medicine, 108 (31.2 %) from the college of AHS and 52 (15 %) from the college of nursing. 118 (34.1%) of the students were from the 2nd year, 38 (11%) were from the 3rd year, 92 (26.6%) were from the 4th year, 42 (12.1%) were from the 5th year and 56 (16.2%) were from the 6th year. 110 (31.8 %) of the respondants had a pharmacist among their family members and 143 (41.3 %) of the respondants had a doctor (Physician) among their family members. 

*Students’ perception and attitude*

Table 2 shows the data obtained by student respondents who gave their opinions on the statements included in the questionnaire. 174 (50.3%) of the students previously heard about the clinical pharmacy program while 272 (78.6%) believed that the clinical pharmacist is an important and integral part of the medical team. When the students were asked if they think that the clinical pharmacist can improve the quality of medical care in a hospital setting, 262 (75.7 %) of them agreed with the statement. The majority of the respondents (246, 71.1 %) stated that the clinical pharmacists as part of medical teams is essential for hospital accreditation. 197 (56.9%) of the students thought that the clinical pharmacist can acquire training in certain medical areas enabling them to perform patient counseling. However, only 137 (39.6 %) of the students reported that there is increasing interest in clinical pharmacy as a profession in KSA. About 60% of the students agreed that doctors and other healthcare staff will accept the involvement of clinical pharmacists in patient management and providing extra services within the framework of clinical pharmacy. Also, about three quarters of the students stated that implication of the clinical pharmacy services will have a positive impact on the management of cancer. 

Interestingly, Table 2 shows that the college of the respondents significantly affected (p < 0.05) the responses of the participants to the statement that the clinical pharmacist is an important and integral part of the medical team. Also, it significantly affected (p < 0.05) the responses of the students to the statements that the clinical pharmacist can acquire training in certain medical areas enabling them to perform patient counseling and that doctors and other healthcare staff will accept the involvement of clinical pharmacists in patient management and providing extra services within the framework of clinical pharmacy.

*Healthcare providers’ demographic data*

Table 3 shows the demographic data of HCPs who gave their opinions in response to the questions included in the questionnaire. The respondents were 84 (44 %) males and 107 (56 %) females. 95 (49.7 %) of the respondents were less than 30 years old, 58 (30.4 %) of the respondents were between 30 and 40 years old and 38 (19.9 %) were above 40 years old. 111 (58.1 %) of the respondents were Saudi while 80 (41.9 %) were non-Saudi. Regarding to respondents’ professions, 62 (32.5 %) were physicians, 68 (35.6 %) were nurses, 17 (8.9 %) were from the physiotherapy department, 25 (13.1 %) were from the laboratory department and 19 (9.9 %) were from the radiology department. Of the total, 12 (6.3 %) reported that they obtained their first professional degree or qualification from either USA or Europe while 110 (57.6 %) of the respondents stated that their first professional qualification was acquired in KSA.

*Healthcare providers’ perception and attitude*

Table 4 shows the data obtained by HCPs who gave their opinions on the statements included in the questionnaire. More than half of HCPs (110, 57.5%) reported that there is a strong healthcare professionals’ willingness to cooperate with the clinical pharmacists. About three quarters of HCPs (137, 71.7%) thought that the clinical pharmacist can improve the quality of patient care in a hospital setting. About two-thirds of the participants believe that the clinical pharmacist is an important integral part of the clinical ward team. Also, about half of the participants reported that the clinical pharmacist can acquire training in certain medical areas to perform patient counseling. Moreover, 115 (60.2%) of HCPs believe that the clinical pharmacist in a clinical ward team is a requirement for hospital accreditation. Also, 130 (68.1%) thought that the clinical pharmacist is able to minimize medication error and improve patient therapy outcomes. Of the participants in the present study, 92 (48.2%) of HCPs believed that the presence of the clinical pharmacist in a clinical ward team will improve the quality of patient care in a hospital setting. However, only 75 (39.3%) of the participants reported that there is increased interest in clinical pharmacy services in KSA. Also, 82 (42.9%) of the participants believed that clinical pharmacy representation in therapeutic policy committee and clinical ward rounds is desirable. Of the participants, 91 (47.6%) thought that the clinical pharmacist has a role in patient medication education. About 60 % of HCPs agreed to the statement that implication of the clinical pharmacy services will have a positive impact on management of cancer. Less than 30% of HCPs believed that the clinical pharmacist has fulfilled his/her role in KSA. Also, only 60 (31.4%) of the participants reported that they have a clinical pharmacist in their institutions. 

Interestingly, table 4 shows that the profession of the respondents significantly affected (p < 0.05) the responses of the participants to the statement that HCPs are willing to cooperate with the clinical pharmacist. Also, it significantly affected (p < 0.05) the responses of HCPs to the statements that the clinical pharmacist can improve the quality of patient care in a hospital setting and the clinical pharmacist can acquire training in certain medical areas to perform patient counseling.

## Discussion

In the last years, significant changes took place in the practice of pharmacy which necessitate changes in procedures and training of pharmacists (AlRuthia et al., 2018; Barnett and Matthews, 2009). Moreover, these changes require more imaginative use of pharmacy skills and involvement of the clinical pharmacists at the healthcare process at all stages (Gans and Maine, 2001; Steckler et al., 2017; Thompson et al., 2018). However, there is still a wide gap between the clinical pharmacists and other HCPs which may deprive the society from getting the benefits of clinical pharmacy in the healthcare process (Al-Aqeel, 2018; Giannitrapani et al., 2018). 

In the present study, there was a strong belief among students of health-related science colleges that clinical pharmacists represent an important integral part of the clinical team and are able to minimize medication errors in the hospital settings. The present study found that only about half of the students heard about the clinical pharmacy in their institutions during their study period. This may be due to absence of patient-oriented PharmD programs (Baniasadi et al., 2014; Bryant et al., 2009; Katoue, 2018; Stemer and Lemmens-Gruber, 2011). Also, a large proportion of the students agreed that HCPs will accept the pharmacists to provide additional services within the framework of clinical pharmacy. This may be attributed to the presence of pharmacists or physicians in the families of these students who gave them this concept (Cohen et al., 2009; Hamblin et al., 2012; Talasaz, 2012).

In the present study, about 40% of the students reported that there is increased interest in clinical pharmacy as a profession in KSA. This may attract the attention of the healthcare authorities to the strong need to apply and expand the role of the clinical pharmacists in the healthcare team to provide the patients with better pharmaceutical care (Almazrou et al., 2015; Nigro et al., 2014; Rafie et al., 2014). This may be in the same line with a previous study in KSA which reported that there is shortage of clinical pharmacists services in most ministry of health hospitals, which necessitates the presence of clinically-oriented training programs for pharmacists and pharmacy students to overcome this shortage and to improve the pharmacists’ role in providing the patients with healthcare in the hospital settings (Albekairy et al., 2015; Alhadad et al., 2016).

In the present study, the college of the respondents significantly affected the responses of the participants to the statement that the clinical pharmacist is an important and integral part of the medical team. Also, it significantly affected the responses of the students to the statements that the clinical pharmacist can acquire training in certain medical areas enabling them to perform patient counseling and that doctors and other healthcare staff will accept the involvement of the clinical pharmacists in patient management and providing extra services within the framework of clinical pharmacy. This may reflect that the students of the college of medicine have more perception and attitude towards the participation of the clinical pharmacy services in the patient’s healthcare than the students of the other health-related science colleges. This may be attributed to the content of the curricula of the subjects given to these students during the college years which may give them a positive attitude towards the participation of the clinical pharmacists in the healthcare team (Abu-Gharbieh et al., 2010; Almeman and Al-Jedai, 2016; Jokanovic et al., 2017).

In the present study, HCPs in Taif hospitals showed a high perception of the role of the clinical pharmacists in improving the therapeutic outcomes of the patients. Also, HCPs in the present study expected that clinical pharmacists can play an important role in direct patient care, particularly in patient counseling and education. Moreover, nearly about three-quarters of HCPs in the present study stated that clinical pharmacists can be of great help to improve the quality of patient care in the hospital settings. This was in agreement with the results of a previous study which indicated that clinical pharmacists working in hospitals managed by international institutions had the ability to provide more efficient clinical pharmacy services because they are involved in review of patient medications and practice an active role in therapy management (Dalton and Byrne, 2017; Ma et al., 2010).

In the present study, HCPs particularly physicians and nurses supported the participation of the clinical pharmacists in the clinical ward team work. They expected that in the near future, they will have the ability to regularly seek advice with regard to patient medications from the clinical pharmacists in their institutions. These findings reinforce the importance of implication of the role of the clinical pharmacists in various aspects of healthcare and prove that the expected resistance of HCPs to implication of the role of the clinical pharmacists should not stand as an obstacle to instituting clinical pharmacy services in the hospital settings (Santos Júnior et al., 2018).

In the present study, Less than 30% of HCPs believed that the clinical pharmacist has fulfilled his/her role in KSA. This may be due to lack of the clinical pharmacists in the hospitals (AlRuthia et al., 2018) which was supported by the findings of the present study where only 31.4% of the participants reported that they have a clinical pharmacist in their institutions. This lack is not limited to the hospitals of the ministry of health but also extends to the private hospitals. Private hospitals should improve the patient’s care services provided by them by incorporating the clinical pharmacists to become a part of their clinical ward teams (Almeman and Al-Jedai, 2016). Also, findings from the present study supported the hypothesis that the clinical pharmacists should perform specific duties that may have a great impact on the healthcare services such as education of the patients and minimizing medication errors (Almaghaslah et al., 2019).

In the present study, the profession of the HCPs significantly affected their response to the statement that HCPs are willing to cooperate with the clinical pharmacist. Also, it significantly affected the responses of HCPs to the statements that the clinical pharmacist can improve the quality of patient care in the hospital settings and the clinical pharmacist can acquire training in certain medical areas to perform patient counseling. This was in agreement with Bondesson et al., (2012) and Sabry and Farid (2014) who reported there is a high level of acceptance by the physicians more than other HCPs to the involvement of the clinical pharmacists in the healthcare team. However, Sabry and Farid (2014) suggested that greater efforts are needed in the Arab countries to increase the physicians’ awareness and knowledge of the importance of the clinical pharmacists in the hospital settings and to promote the benefits from the clinical pharmacy services.

In the present study, about three quarters of the students and 60% of HCPs agreed to the statement that implication of the clinical pharmacy services will have a positive impact on management of cancer. This was in the same line with Delpeuch et al., (2015) and Yokoyama et al., (2018) who reported that patients who received medical care from a collaborative team that included a clinical pharmacist showed significant improvement in the most important key indicators of cancer management. 

On the other side, some studies reported resistance of HCPs especially the physicians to implication of the role of the clinical pharmacists in the hospital settings (Perera et al., 2011). This resistance may be attributed to the lack of direct contact between the physicians’ and the clinical pharmacists participating in the clinical activities (Truong et al., 2017). Also, lack of knowledge with the importance of the clinical pharmacists in healthcare and deficiency of well-trained and efficient clinical pharmacists in the hospitals may be contributing factors (Albekairy et al., 2015). In order to overcome this resistance, continuous improvement and incorporation of courses related to inter-professional relationships between HCPs and the clinical pharmacists in the curricula of the medical, pharmaceutical and nursing education should be applied to encourage the collaboration between the clinical pharmacists and other HCPs in provision of patient care (Aljadhey et al., 2016).

In conclusion, HCPs and the students of health-related science colleges in this study valued the role of the clinical pharmacists in healthcare delivery and management of cancer. However, new developments in clinical pharmacy services in Taif hospitals are recommended to improve the perceptions and attitudes towards the services of clinical pharmacy and improve the healthcare provided to the patients. Also, well-organized programs should be included in the curricula of the subjects given to the students of health-related science colleges to improve their perception and attitude towards the clinical pharmacy services. Further studies are needed to investigate the impact of improving the clinical pharmacy services on the outcomes of cancer management. 

**Table 1:** Demographic Data of the Students

Demographic information	Number (%)
Gender:	
Male	79 (22.8 %)
Female	267 (77.2 %)
Age:	
Less than 20 years	83 (24%)
From 20 to 25 years	258 (74.6 %)
More than 25 years	5 (1.4%)
College:	
Medicine	186 (53.8 %)
Applied Health Sciences	108 (31.2 %)
Nursing	52 (15 %)
Education level:	
2^nd^ year	118 (34.1%)
3^rd^ year	38 (11%)
4^th^ year	92 (26.6%)
5^th^ year	42 (12.1%)
6^th^ year	56 (16.2%)
Presence of a pharmacist among family members	
Yes	110 (31.8 %)
No	236 (68.2%)
Presence of a doctor (Physician) among family members	
Yes	143 (41.3%)
No	203 (58.7%)

**Table 2:** Data Obtained by Student Respondents who Gave Their Opinions on the Statements Included in the Questionnaire (N = 346)

Questions	Agree n (%)	Neutral n (%)	Disagree n (%)	p-value
1. Previously heard about clinical pharmacy programs	174 (50.3 )	67 (19.4)	105 (30.3)	0.29
2. The clinical pharmacist is an important and integral part of the medical team	272 (78.6)	27 (7.8)	47 (13.6)	<0.001
3. The clinical pharmacist can improve the quality of medical care in a hospital setting	262 (75.7)	32 (9.2)	52 (15)	0.32
4. Clinical pharmacists as part of medical teams is essential for hospital accreditation	246 (71.1)	46 (13.3)	54 (15.6)	0.06
5. The clinical pharmacist can acquire training in certain medical areas enabling them to perform patient counseling	197 (56.9)	83 (24)	66 (19.1)	0.008
6. There is increasing interest in clinical pharmacy as a profession in KSA	137 (39.6)	116 (33.5)	93 (26.9)	0.21
7. Doctors and other healthcare staff will accept the involvement of clinical pharmacists in patient management and providing extra services within the framework of clinical pharmacy	208 (60.1)	80 (23.1)	58 (16.8)	0.007
8. Implication of the clinical pharmacy services will have a positive impact on management of cancer	251 (72.5)	42 (12.1)	53 (15.4)	0.08

**Table 3:** Demographic Data of Healthcare Providers

Demographic information	Number (%)
Gender:	
Male	84 (44 %)
Female	107 (56 %)
Age:	
Below 30 years	95 (49.7 %)
30 – 40 years	58 (30.4 %)
More than 40 years	38 (19.9 %)
Nationality:	
Saudi	111 (58.1 %)
Non-Saudi	80 (41.9 %)
Profession:	
Physician	62 (32.5 %)
Nurse	68 (35.6 %)
Physiotherapy department	17 (8.9 %)
Laboratory department	25 (13.1 %)
Radiology department	19 (9.9 %)
Professionally qualified in:	
USA/Europe	12 (6.3 %)
KSA	110 (57.6 %)
Others	69 (36.1 %)

**Table 4:** Data Obtained by Hcps Who Gave Their Opinions on the Statements Included in the Questionnaire (N = 191).

Questions	HCPs	Agree	Neutral	Disagree	p-value
1. Healthcare professionals’ willingness to cooperate with the clinical pharmacist	Physician	43	14	5	<0.001
	Nurse	51	12	5	
	Physiotherapy department	5	7	5	
	Laboratory department	6	13	6	
	Radiology department	5	9	5	
	Total	110 (57.5%)	55 (28.8%)	26 (13.6%)	
2. The clinical pharmacist can improve the quality of patient care in a hospital setting	Physician	51	6	5	<0.001
	Nurse	54	5	9	
	Physiotherapy department	12	0	5	
	Laboratory department	11	8	6	
	Radiology department	9	5	5	
	Total	137 (71.7%)	24 (12.6%)	30 (15.7%)	
3. The clinical pharmacist is an important integral part of the clinical ward team	Physician	42	12	8	0.54
	Nurse	44	13	11	
	Physiotherapy department	9	5	3	
	Laboratory department	20	3	2	
	Radiology department	9	5	5	
	Total	124 (64.9%)	38 (19.9%)	29 (15.2%)	
4. The clinical pharmacist can acquire training in certain medical areas to perform patient counseling	Physician	41	16	5	<0.001
	Nurse	44	20	4	
	Physiotherapy department	6	10	1	
	Laboratory department	5	19	1	
	Radiology department	5	13	1	
	Total	101 (52.9%)	78 (40.8%)	12 (6.3%)	
5. The clinical pharmacist in a clinical ward team is a requirement for hospital accreditation	Physician	36	22	4	0.74
	Nurse	44	19	5	
	Physiotherapy department	9	7	1	
	Laboratory department	15	10	0	
	Radiology department	11	8	0	
	Total	115 (60.2%)	66 (34.6%)	10 (5.2%)	
6. The clinical pharmacist is able to minimize medication errors and improve patient therapy outcomes	Physician	39	20	3	0.8
	Nurse	49	17	2	
	Physiotherapy department	11	5	1	
	Laboratory department	20	5	0	
	Radiology department	11	7	1	
	Total	130 (68.1%)	54 (28.3%)	7 (3.7%)	
7. There is increased interest in clinical pharmacy services in KSA	Physician	25	30	7	0.43
	Nurse	26	35	7	
	Physiotherapy department	4	13	0	
	Laboratory department	13	9	3	
	Radiology department	7	9	3	
	Total	75 (39.3%)	96 (50.3%)	20 (10.4%)	
8. Clinical pharmacy representation in therapeutic policy committee and clinical ward rounds is desirable	Physician	25	34	2	0.32
	Nurse	26	40	2	
	Physiotherapy department	8	7	2	
	Laboratory department	13	10	3	
	Radiology department	10	9	0	
	Total	82 (42.9%)	100 (52.4%)	9 (4.7%)	
	HCPs	Agree	Neutral	Disagree	P-value
	Physician	31	30	1	0.79
	Nurse	30	37	1	
	Physiotherapy department	10	6	1	
	Laboratory department	11	14	0	
	Radiology department	9	10	0	
	Total	91 (47.6%)	97 (50.8%)	3 (1.6%)	
	Physician	15	25	22	0.38
	Nurse	18	30	20	
	Physiotherapy department	3	8	6	
	Laboratory department	9	10	6	
	Radiology department	10	6	3	
	Total	55 (28.8%)	79 (41.4%)	57 (29.8%)	
	Physician	28	34	0	0.82
	Nurse	33	33	2	
	Physiotherapy department	8	8	1	
	Laboratory department	13	11	1	
	Radiology department	10	9	0	
	Total	92 (48.2%)	95 (49.7%)	4 (2.1%)	
	Physician	15	0	47	0.2
	Nurse	27	0	41	
	Physiotherapy department	7	0	10	
	Laboratory department	5	0	20	
	Radiology department	6	0	13	
	Total	60 (31.4%)	0	131 (68.6%)	
	Physician	34	7	21	0.3
	Nurse	52	5	11	
	Physiotherapy department	6	3	8	
	Laboratory department	12	4	9	
	Radiology department	11	3	5	
	Total	115 (60.2%)	22 (11.5%)	54 (28.3%)	
